# The genome sequence of the virgin bagworm, *Luffia ferchaultella *(Stephens, 1850)

**DOI:** 10.12688/wellcomeopenres.23768.1

**Published:** 2025-02-26

**Authors:** Samuel Whiteford, Wellcome Sanger Institute Tree of Life Management, Samples and Laboratory team, Wellcome Sanger Institute Scientific Operations: Sequencing Operations, Wellcome Sanger Institute Tree of Life Core Informatics team, Tree of Life Core Informatics collective, Darwin Tree of Life Consortium

**Affiliations:** 1 Department of Evolution, Ecology and Behaviour, University of Liverpool, Liverpool, England, UK

**Keywords:** Luffia ferchaultella, virgin bagworm, genome sequence, chromosomal, Lepidoptera

## Abstract

We present a genome assembly from an individual female *Luffia ferchaultella* (the Virgin Bagworm; Arthropoda; Insecta; Lepidoptera; Psychidae). The genome sequence spans 645.30 megabases. Most of the assembly is scaffolded into 31 chromosomal pseudomolecules, including the Z sex chromosome. The mitochondrial genome has also been assembled and is 15.37 kilobases in length. Gene annotation of this assembly on Ensembl identified 12,416 protein-coding genes.

## Species taxonomy

Eukaryota; Opisthokonta; Metazoa; Eumetazoa; Bilateria; Protostomia; Ecdysozoa; Panarthropoda; Arthropoda; Mandibulata; Pancrustacea; Hexapoda; Insecta; Dicondylia; Pterygota; Neoptera; Endopterygota; Amphiesmenoptera; Lepidoptera; Glossata; Neolepidoptera; Heteroneura; Ditrysia; Tineoidea; Psychidae; Psychinae; *Luffia*; *Luffia ferchaultella* (Stephens, 1850) (NCBI:txid1870047).

## Background

**Figure 1.**
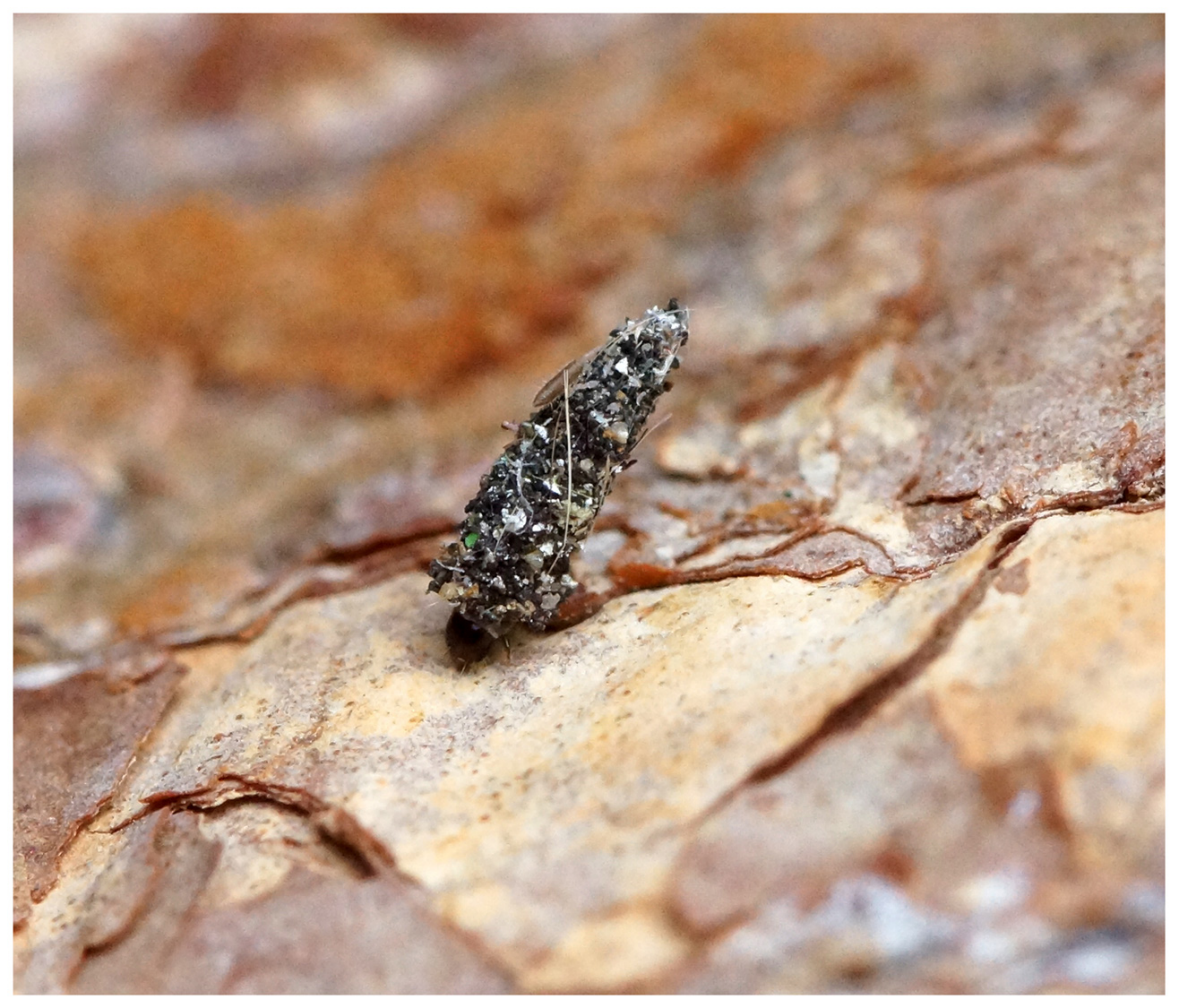
Photograph of a *Luffia ferchaultella* larva by Ben Sale (not the specimen used for genome sequencing).

The Psychid moth *Luffia ferchaultella* ( Figure 1) is locally abundant in parts of the United Kingdom and Western Europe ( McDonogh, 1939; Narbel-Hofstetter, 1964). Most of an individual’s lifespan is spent in the larval stages on trees and rock surfaces in large colonies. Wind-dispersal occurs at early instars, aided by silk thread. Once migrated, larvae feed on lichens and algae and become visually cryptic, due to an endearing protective bag construction behaviour ( McDonogh, 1939). The larval stage exhibits developmental sensitivity to anthropogenic emissions, which has been utilised for biomonitoring purposes ( Sims *et al.*, 2009; Sims *et al.*, 2011).

At the final instar, larvae pupate within the protective bag and subsequently emerge as a short-lived adult form. The adult moth is morphologically neotenous and apterous. It reproduces asexually by thelytokous parthenogenesis, laying eggs into the vacated larval case. The overall similarity of these organisms to females of the sexually-reproducing species *Luffia lapidella*, in addition to its ability to hybridise, has led to suggestions that *L. ferchaultella* may be a derived parthenogenetic form of *L. lapidella* ( Narbel-Hofstetter, 1963).

*Luffia ferchaultella* is noteworthy for its apparent geographical diversity in the parthenogenetic mechanism by which the diploid chromosome number is restored. This variation is shown to be consistent within broods ( Narbel-Hofstetter, 1965). Further work also showed a variety of sex development outcomes in crosses between *L. ferchaultella* and its sister species ( Narbel-Hofstetter, 1966). In addition to an assembled host genome, the data presented here also reveals the presence of the endosymbiont *Wolbachia,* which is known to influence arthropod chromosome dynamics to induce parthenogenesis; however, a causative link has not been established in this species ( Fricke & Lindsey, 2023; Kern *et al.*, 2015).

Psychidae moths display an ancestral ZZ/Z0 sex chromosome karyotype, in contrast to the ZZ/ZW system more commonly observed in the ditrysian moths ( Hejníčková *et al.*, 2019). The genome sequence of *L. ferchaultella* may therefore help generate insights into the variability of sex-determination systems, the complexity of host-endosymbiont conflicts and parthenogenesis observed in the Lepidoptera.

Here we present a chromosome-level genome sequence for *Luffia ferchaultella*, based on a female specimen from Liverpool, England, UK, sequenced as part of the Darwin Tree of Life Project.

## Genome sequence report

The genome of a female *Luffia ferchaultella* larva was sequenced using Pacific Biosciences single-molecule HiFi long reads, generating a total of 20.65 Gb (gigabases) from 1.97 million reads, providing approximately 30-fold coverage. Primary assembly contigs were scaffolded with chromosome conformation Hi-C data, which produced 122.54 Gb from 811.53 million reads. Specimen and sequencing details are provided in Table 1.

**Table 1.** Specimen and sequencing data for *Luffia ferchaultella*.

Project information			
**Study title**	Luffia ferchaultella (virgin bagworm)		
**Umbrella BioProject**	PRJEB59780		
**Species**	*Luffia ferchaultella*		
**BioSample**	SAMEA9252612		
**NCBI taxonomy ID**	1870047		
Specimen information			
**Technology**	**ToLID**	**BioSample accession**	**Organism part**
**PacBio long read sequencing**	ilLufFerc1	SAMEA9252618	Whole organism
**Hi-C sequencing**	ilLufFerc3	SAMEA9252620	Whole organism
**RNA sequencing**	ilLufFerc4	SAMEA9252621	Whole organism
Sequencing information			
**Platform**	**Run accession**	**Read count**	**Base count (Gb)**
**Hi-C Illumina NovaSeq 6000**	ERR10890726	8.12e+08	122.54
**PacBio Sequel IIe**	ERR10879928	1.97e+06	20.65
**RNA Illumina NovaSeq 6000**	ERR11242526	7.12e+07	10.76

**Figure 2.**
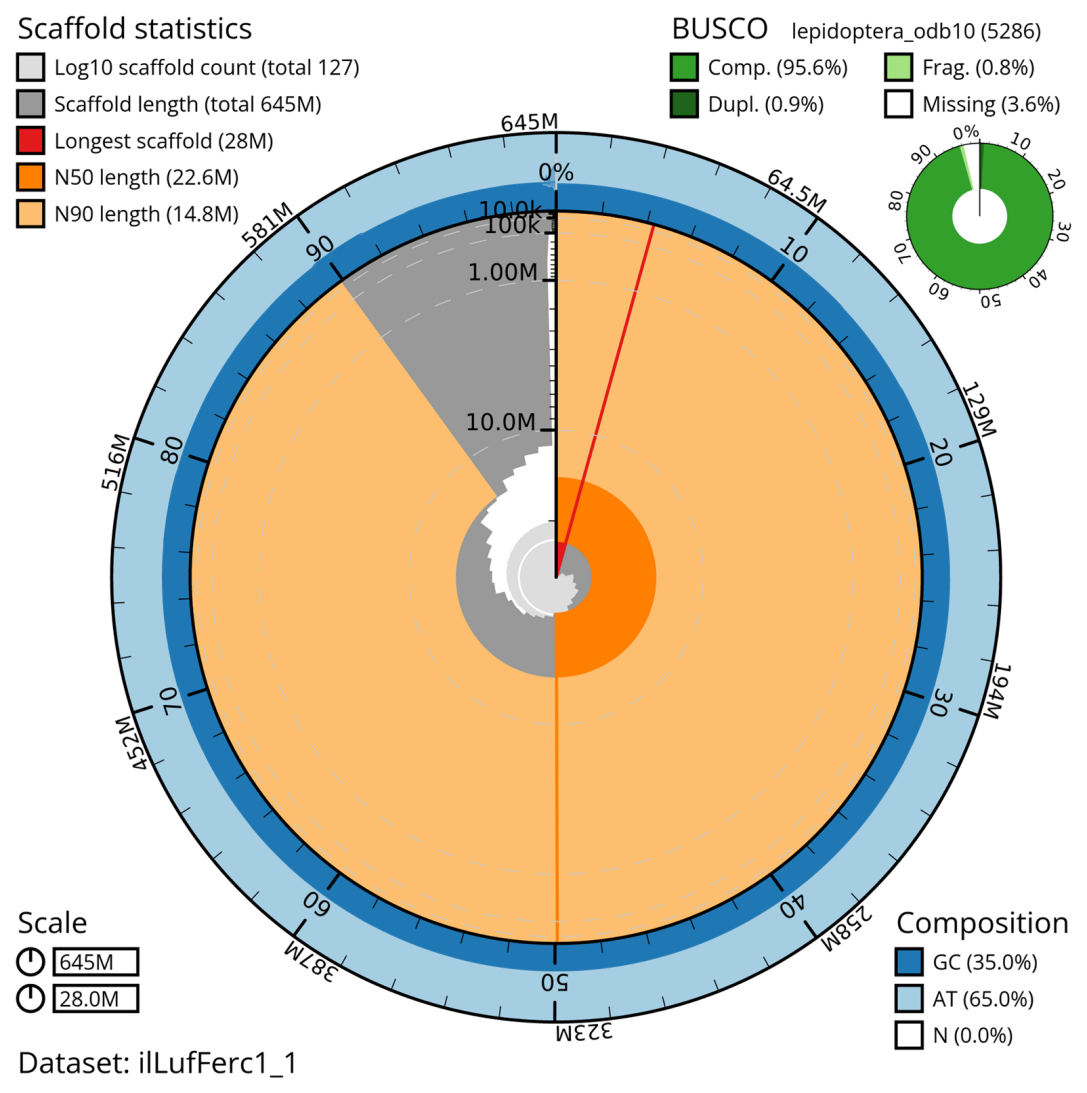
Genome assembly of *Luffia ferchaultella*, ilLufFerc1.1: metrics. The BlobToolKit snail plot shows N50 metrics and BUSCO gene completeness. The main plot is divided into 1,000 bins around the circumference with each bin representing 0.1% of the 645,276,354 bp assembly. The distribution of scaffold lengths is shown in dark grey with the plot radius scaled to the longest scaffold present in the assembly (27,956,967 bp, shown in red). Orange and pale-orange arcs show the N50 and N90 scaffold lengths (22,599,242 and 14,756,327 bp), respectively. The pale grey spiral shows the cumulative scaffold count on a log scale with white scale lines showing successive orders of magnitude. The blue and pale-blue area around the outside of the plot shows the distribution of GC, AT and N percentages in the same bins as the inner plot. A summary of complete, fragmented, duplicated and missing BUSCO genes in the lepidoptera_odb10 set is shown in the top right. An interactive version of this figure is available at https://blobtoolkit.genomehubs.org/view/ilLufFerc1_1/dataset/ilLufFerc1_1/snail.

**Figure 3.**
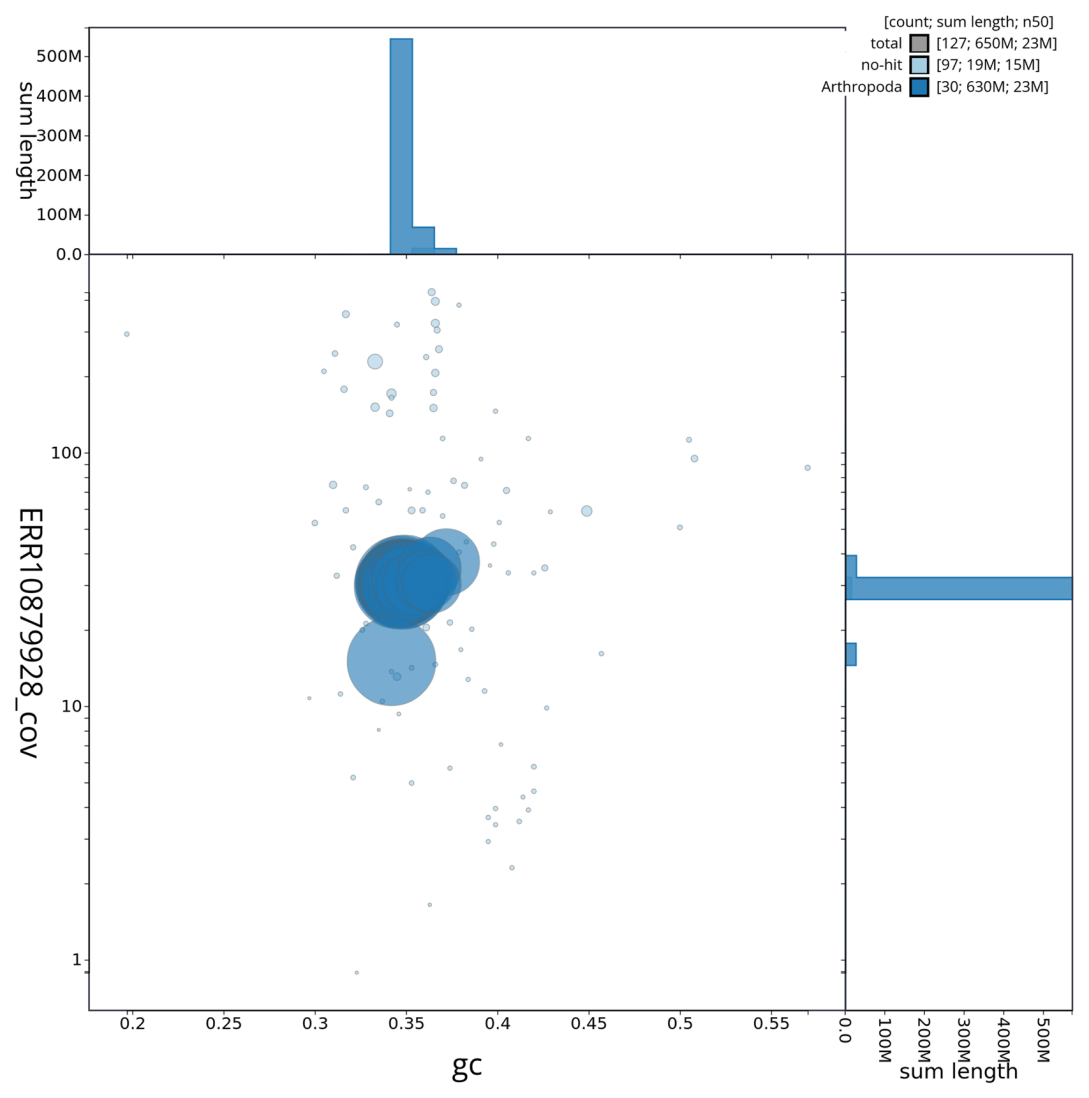
Genome assembly of *Luffia ferchaultella*, ilLufFerc1.1: BlobToolKit GC-coverage plot. Sequences are coloured by phylum. Circles are sized in proportion to sequence length. Histograms show the distribution of sequence length sum along each axis. An interactive version of this figure is available at https://blobtoolkit.genomehubs.org/view/ilLufFerc1_1/dataset/ilLufFerc1_1/blob.

**Figure 4.**
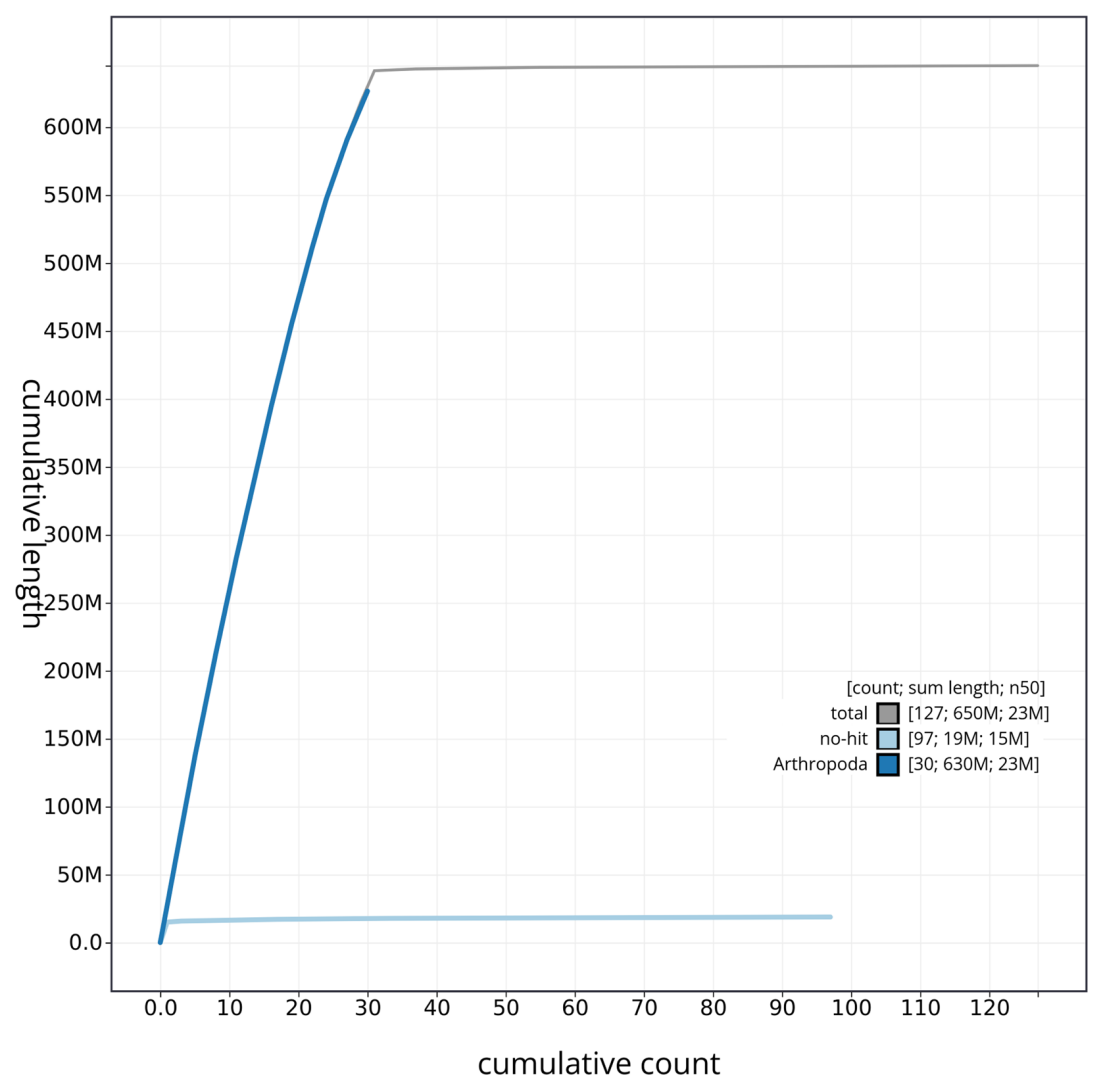
Genome assembly of *Luffia ferchaultella* ilLufFerc1.1: BlobToolKit cumulative sequence plot. The grey line shows cumulative length for all sequences. Coloured lines show cumulative lengths of sequences assigned to each phylum using the buscogenes taxrule. An interactive version of this figure is available at https://blobtoolkit.genomehubs.org/view/ilLufFerc1_1/dataset/ilLufFerc1_1/cumulative.

Manual assembly curation corrected 76 missing joins or mis-joins and 15 haplotypic duplications, reducing the assembly length by 0.5% and the scaffold number by 13.61%, and increasing the scaffold N50 by 2.33%. The final assembly has a total length of 645.30 Mb in 126 sequence scaffolds with a scaffold N50 of 22.6 Mb ( Table 2). The snail plot in Figure 2 provides a summary of the assembly statistics, while the distribution of assembly scaffolds on GC proportion and coverage is shown in Figure 3. The cumulative assembly plot in Figure 4 shows curves for subsets of scaffolds assigned to different phyla.

**Table 2.** Genome assembly data for *Luffia ferchaultella*, ilLufFerc1.1.

Genome assembly		
Assembly name	ilLufFerc1.1	
Assembly accession	GCA_949709985.1	
*Accession of alternate haplotype*	*GCA_949710065.1*	
Span (Mb)	645.30	
Number of contigs	799	
Contig N50 length (Mb)	1.5	
Number of scaffolds	126	
Scaffold N50 length (Mb)	22.6	
Longest scaffold (Mb)	27.96	
Assembly metrics *		*Benchmark*
Consensus quality (QV)	60.9	*≥ 50*
*k*-mer completeness	98.81% (combined)	*≥ 95%*
BUSCO **	C:95.6%[S:94.7%,D:0.9%], F:0.8%,M:3.6%,n:5,286	*C ≥ 95%*
Percentage of assembly mapped to chromosomes	99.59%	*≥ 95%*
Sex chromosomes	ZO	*localised homologous pairs*
Organelles	Mitochondrial genome: 15.37 kb	*complete single alleles*
Genome annotation of assembly GCA_949709985.1 at Ensembl		
Number of protein-coding genes	12,416	
Number of non-coding genes	1,308	
Number of gene transcripts	21,594	

* Assembly metric benchmarks are adapted from column VGP-2020 of “Table 1: Proposed standards and metrics for defining genome assembly quality” from Rhie *et al.* (2021). ** BUSCO scores based on the lepidoptera_odb10 BUSCO set using version 5.3.2. C = complete [S = single copy, D = duplicated], F = fragmented, M = missing, n = number of orthologues in comparison. A full set of BUSCO scores is available at https://blobtoolkit.genomehubs.org/view/ilLufFerc1_1/dataset/ilLufFerc1_1/busco.

**Figure 5.**
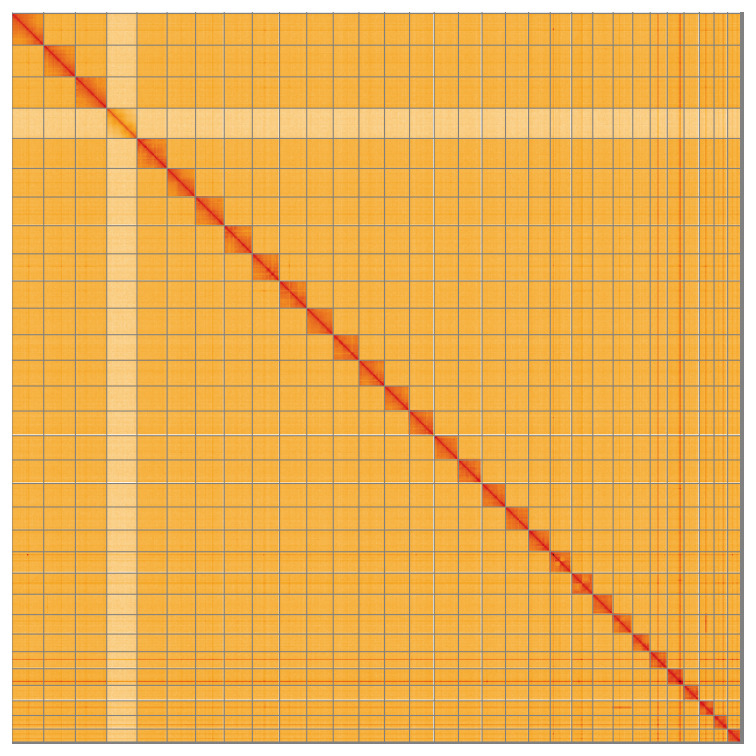
Genome assembly of *Luffia ferchaultella* ilLufFerc1.1: Hi-C contact map of the ilLufFerc1.1 assembly, visualised using HiGlass. Chromosomes are shown in order of size from left to right and top to bottom. An interactive version of this figure may be viewed at https://genome-note-higlass.tol.sanger.ac.uk/l/?d=bcbTo49NQpi_wxtfcscOZw.

Most (99.59%) of the assembly sequence was assigned to 31 chromosomal-level scaffolds, representing 30 autosomes and the Z sex chromosome. The specimen is a ZO female. Chromosome-scale scaffolds confirmed by the Hi-C data are named in order of size ( Figure 5; Table 3).

**Table 3.** Chromosomal pseudomolecules in the genome assembly of *Luffia ferchaultella*, ilLufFerc1.

INSDC accession	Name	Length (Mb)	GC%
OX453240.1	1	27.96	34.5
OX453241.1	2	27.9	35.0
OX453242.1	3	27.55	35.0
OX453244.1	4	26.46	35.0
OX453245.1	5	25.22	34.5
OX453246.1	6	25.21	34.5
OX453247.1	7	24.68	34.5
OX453248.1	8	23.98	34.5
OX453249.1	9	23.9	34.5
OX453250.1	10	23.33	34.5
OX453251.1	11	22.63	34.5
OX453252.1	12	22.6	35.0
OX453253.1	13	22.08	35.0
OX453254.1	14	21.66	34.5
OX453255.1	15	21.31	35.0
OX453256.1	16	20.78	35.0
OX453257.1	17	20.72	35.0
OX453258.1	18	20.26	34.5
OX453259.1	19	18.88	35.0
OX453260.1	20	19.13	35.0
OX453261.1	21	18.53	35.5
OX453262.1	22	17.97	35.0
OX453263.1	23	17.17	35.0
OX453264.1	24	15.4	35.0
OX453265.1	25	14.76	37.0
OX453266.1	26	15.02	35.5
OX453267.1	27	13.51	35.5
OX453268.1	28	12.93	36.5
OX453269.1	29	11.97	36.0
OX453270.1	30	11.39	36.5
OX453243.1	Z	26.64	34.0
OX453271.1	MT	0.02	20.0

While not fully phased, the assembly deposited is of one haplotype. Contigs corresponding to an alternate haplotype have also been deposited. The mitochondrial genome was also assembled and can be found as a contig within the multifasta file of the genome submission.

The estimated Quality Value (QV) of the final assembly is 60.9. The *k*-mer completeness for the combined primary assembly and alternate haplotypes was 98.81% (primary 83.55%; alternate 73.97%). The assembly achieves a BUSCO v5.3.2 completeness of 95.6% (single = 94.7%, duplicated = 0.9%), using the lepidoptera_odb10 reference set ( *n* = 5,286).

## Genome annotation report

The *Luffia ferchaultella* genome assembly (GCA_949709985.1) was annotated at the European Bioinformatics Institute (EBI) on Ensembl Rapid Release. The resulting annotation includes 21,594 transcribed mRNAs from 12,416 protein-coding and 1,308 non-coding genes ( Table 2; https://rapid.ensembl.org/Luffia_ferchaultella_GCA_949709985.1/Info/Index). The average transcript length is 20,560.78. There are 1.57 coding transcripts per gene and 7.40 exons per transcript.

## Methods

### Sample acquisition

Larval specimens of *Luffia ferchaultella* were hand-collected from Liverpool, England, UK (latitude 53.39, longitude –2.89) on 2021-04-20. The specimens were collected and identified by Samuel Whiteford (University of Liverpool) and then killed and stored in a –80 °C freezer. One of the specimens (specimen ID SAN0001394, ToLID ilLufFerc1) was used for PacBio HiFi sequencing, a second specimen (specimen ID SAN0001396, ToLID ilLufFerc3) was used for Hi-C scaffolding, and a third specimen (specimen ID SAN0001397, ToLID ilLufFerc4) was used for RNA sequencing.

### Nucleic acid extraction

The workflow for high molecular weight (HMW) DNA extraction at the Wellcome Sanger Institute (WSI) Tree of Life Core Laboratory includes a sequence of procedures: sample preparation; sample homogenisation, DNA extraction, fragmentation, and clean-up. In sample preparation, the ilLufFerc1 sample was weighed and dissected on dry ice ( Jay *et al.*, 2023).

Tissue from whole organism was homogenised using a Power-Masher II tissue disruptor ( Denton *et al.*, 2023). HMW DNA was extracted using the Automated MagAttract v1 protocol ( Sheerin *et al.*, 2023). For ULI PacBio sequencing, DNA was fragmented using the Covaris g-TUBE method ( Oatley *et al.*, 2023). Sheared DNA was purified by solid-phase reversible immobilisation ( Strickland *et al.*, 2023), using AMPure PB beads to eliminate shorter fragments and concentrate the DNA. The concentration of the sheared and purified DNA was assessed using a Nanodrop spectrophotometer and Qubit Fluorometer using the Qubit dsDNA High Sensitivity Assay kit. Fragment size distribution was evaluated by running the sample on the FemtoPulse system.

RNA was extracted from whole organism tissue of ilLufFerc4 in the Tree of Life Laboratory at the WSI using the RNA Extraction: Automated MagMax™ *mir* Vana protocol ( do Amaral *et al.*, 2023). The RNA concentration was assessed using a Nanodrop spectrophotometer and a Qubit Fluorometer using the Qubit RNA Broad-Range Assay kit. Analysis of the integrity of the RNA was done using the Agilent RNA 6000 Pico Kit and Eukaryotic Total RNA assay.

### Hi-C sample preparation

Tissue from the ilLufFerc3 sample was processed using the Arima-HiC v2 kit at the WSI Scientific Operations core. In brief, 20–50 mg of frozen tissue (stored at –80 °C) was fixed, and the DNA crosslinked using a TC buffer with 22% formaldehyde concentration. After crosslinking the tissue was homogenised using the Diagnocine Power Masher-II and BioMasher-II tubes and pestles. Following the Arima-HiC v2 kit manufacturer's instructions, crosslinked DNA was digested using a restriction enzyme master mix. The 5’-overhangs were filled in and labelled with biotinylated nucleotides and proximally ligated. An overnight incubation was carried out for enzymes to digest remaining proteins and for crosslinks to reverse. A clean up was performed with SPRIselect beads prior to library preparation. Additionally, the biotinylation percentage was estimated using the Qubit Fluorometer v4.0 (Thermo Fisher Scientific) and Qubit HS Assay Kit and Arima-HiC v2 QC beads.

### Library preparation and sequencing

Library preparation and sequencing were performed at the WSI Scientific Operations core.

***PacBio Hi-Fi***

Ultra-low input libraries were prepared using PacBio SMRTbell® Express Template Prep Kit 2.0 and PacBio SMRTbell® gDNA Sample Amplification Kit. To begin, samples were normalised to 20 ng of DNA. Initial removal of single-strand overhangs, DNA damage repair, and end repair/A-tailing were performed per manufacturer’s instructions. From the SMRTbell® gDNA Sample Amplification Kit, amplification adapters were then ligated. A 0.85X pre-PCR clean-up was performed with Promega ProNex beads and the sample was then divided into two for a dual PCR. PCR reactions A and B each followed the PCR programs as described in the manufacturer’s protocol. A 0.85X post-PCR clean-up was performed with ProNex beads for PCR reactions A and B and DNA concentration was quantified using the Qubit Fluorometer v2.0 (Thermo Fisher Scientific) and Qubit HS Assay Kit and fragment size analysis was carried out using the Agilent Femto Pulse Automated Pulsed Field CE Instrument (Agilent Technologies) and gDNA 55kb BAC analysis kit. PCR reactions A and B were then pooled, ensuring the total mass was ≥500 ng in 47.4 μl. The pooled sample then repeated the process for DNA damage repair, end repair/A-tailing and additional hairpin adapter ligation. A 1X clean-up was performed with ProNex beads and DNA concentration was quantified using the Qubit and fragment size analysis was carried out using the Agilent Femto Pulse Automated Pulsed Field CE Instrument (Agilent Technologies). Size selection was performed using Sage Sciences' PippinHT system with target fragment size determined by analysis from the Femto Pulse, usually a value between 4000 and 9000bp. Size selected libraries then had a final 1.0X clean up with ProNex beads and normalised to 2nM before proceeding to sequencing.

Samples were sequenced using the Sequel IIe system (Pacific Biosciences, California, USA). The concentration of the library loaded onto the Sequel IIe at a maximum 80pM. The SMRT link software, a PacBio web-based end-to-end workflow manager, was used to set-up and monitor the run, as well as perform primary and secondary analysis of the data upon completion.

***Hi-C***

For Hi-C library preparation, DNA was fragmented using the Covaris E220 sonicator (Covaris) and size selected using SPRISelect beads to 400 to 600 bp. The DNA was then enriched using the Arima-HiC v2 kit Enrichment beads. Using the NEBNext Ultra II DNA Library Prep Kit (New England Biolabs) for end repair, a-tailing, and adapter ligation. This uses a custom protocol which resembles the standard NEBNext Ultra II DNA Library Prep protocol but where library preparation occurs while DNA is bound to the Enrichment beads. For library amplification, 10-16 PCR cycles were required, determined by the sample biotinylation percentage. The Hi-C sequencing was performed using paired-end sequencing with a read length of 150 bp on an Illumina NovaSeq 6000.

***RNA***

Poly(A) RNA-Seq libraries were constructed using the NEB Ultra II RNA Library Prep kit. RNA sequencing was performed on the Illumina NovaSeq 6000 (RNA-Seq) instruments.

### Genome assembly, curation and evaluation

***Assembly***

The original assembly of HiFi reads was performed using Hifiasm ( Cheng *et al.*, 2021) with the --primary option. Haplotypic duplications were identified and removed with purge_dups ( Guan *et al.*, 2020). Hi-C reads are further mapped with bwamem2 ( Vasimuddin *et al.*, 2019) to the primary contigs, which are further scaffolded using the provided Hi-C data ( Rao *et al.*, 2014) in YaHS ( Zhou *et al.*, 2023) using the --break option. Scaffolded assemblies are evaluated using Gfastats ( Formenti *et al.*, 2022), BUSCO ( Manni *et al.*, 2021) and MERQURY.FK ( Rhie *et al.*, 2020).

The mitochondrial genome was assembled using MitoHiFi ( Uliano-Silva *et al.*, 2023), which runs MitoFinder ( Allio *et al.*, 2020) or MITOS ( Bernt *et al.*, 2013) and uses these annotations to select the final mitochondrial contig and to ensure the general quality of the sequence.

***Assembly curation***

The assembly was decontaminated using the Assembly Screen for Cobionts and Contaminants (ASCC) pipeline (article in preparation). Manual curation was primarily conducted using PretextView ( Harry, 2022), with additional insights provided by JBrowse2 ( Diesh *et al.*, 2023) and HiGlass ( Kerpedjiev *et al.*, 2018). Scaffolds were visually inspected and corrected as described by Howe *et al*. (2021). Any identified contamination, missed joins, and mis-joins were corrected, and duplicate sequences were tagged and removed. The entire process is documented at https://gitlab.com/wtsi-grit/rapid-curation (article in preparation).

***Assembly quality assessment***

The Merqury.FK tool ( Rhie *et al.*, 2020), run in a Singularity container ( Kurtzer *et al.*, 2017), was used to evaluate *k*-mer completeness and assembly quality for the primary and alternate haplotypes using the *k*-mer databases ( *k* = 31) that were computed prior to genome assembly. The analysis outputs included assembly QV scores and completeness statistics.

A Hi-C contact map was produced for the final version of the assembly. The Hi-C reads were aligned using bwa-mem2 ( Vasimuddin *et al.*, 2019) and the alignment files were combined using SAMtools ( Danecek *et al.*, 2021). The Hi-C alignments were converted into a contact map using BEDTools ( Quinlan & Hall, 2010) and the Cooler tool suite ( Abdennur & Mirny, 2020). The contact map is visualised in HiGlass ( Kerpedjiev *et al.*, 2018).

The genome was also analysed within the BlobToolKit environment ( Challis *et al.*, 2020) and BUSCO scores ( Manni *et al.*, 2021) were calculated.

Table 4 contains a list of relevant software tool versions and sources.

**Table 4.** Software tools: versions and sources.

Software tool	Version	Source
BlobToolKit	4.2.1	https://github.com/blobtoolkit/blobtoolkit
BUSCO	5.3.2	https://gitlab.com/ezlab/busco
Hifiasm	0.16.1-r375	https://github.com/chhylp123/hifiasm
HiGlass	1.11.6	https://github.com/higlass/higlass
Merqury	MerquryFK	https://github.com/thegenemyers/MERQURY.FK
MitoHiFi	2	https://github.com/marcelauliano/MitoHiFi
PretextView	0.2.5	https://github.com/wtsi-hpag/PretextView
purge_dups	1.2.3	https://github.com/dfguan/purge_dups
YaHS	yahs-1.1.91eebc2	https://github.com/c-zhou/yahs

### Genome annotation

The Ensembl Genebuild annotation system ( Aken *et al.*, 2016) was used to generate annotation for the *Luffia ferchaultella* assembly (GCA_949709985.1) in Ensembl Rapid Release at the EBI. Annotation was created primarily through alignment of transcriptomic data to the genome, with gap filling via protein-to-genome alignments of a select set of proteins from UniProt ( UniProt Consortium, 2019).

### Wellcome Sanger Institute – Legal and Governance

The materials that have contributed to this genome note have been supplied by a Darwin Tree of Life Partner. The submission of materials by a Darwin Tree of Life Partner is subject to the **‘Darwin Tree of Life Project Sampling Code of Practice’**, which can be found in full on the Darwin Tree of Life website here. By agreeing with and signing up to the Sampling Code of Practice, the Darwin Tree of Life Partner agrees they will meet the legal and ethical requirements and standards set out within this document in respect of all samples acquired for, and supplied to, the Darwin Tree of Life Project.

Further, the Wellcome Sanger Institute employs a process whereby due diligence is carried out proportionate to the nature of the materials themselves, and the circumstances under which they have been/are to be collected and provided for use. The purpose of this is to address and mitigate any potential legal and/or ethical implications of receipt and use of the materials as part of the research project, and to ensure that in doing so we align with best practice wherever possible. The overarching areas of consideration are:

•     Ethical review of provenance and sourcing of the material

•     Legality of collection, transfer and use (national and international)

Each transfer of samples is further undertaken according to a Research Collaboration Agreement or Material Transfer Agreement entered into by the Darwin Tree of Life Partner, Genome Research Limited (operating as the Wellcome Sanger Institute), and in some circumstances other Darwin Tree of Life collaborators.

## Data Availability

European Nucleotide Archive: Luffia ferchaultella (virgin bagworm). Accession number PRJEB59780; https://identifiers.org/ena.embl/PRJEB59780. The genome sequence is released openly for reuse. The *Luffia ferchaultella* genome sequencing initiative is part of the Darwin Tree of Life (DToL) project. All raw sequence data and the assembly have been deposited in INSDC databases. Raw data and assembly accession identifiers are reported in Table 1 and Table 2.

## Author information

Members of the Wellcome Sanger Institute Tree of Life Management, Samples and Laboratory team are listed here: https://doi.org/10.5281/zenodo.12162482.

Members of Wellcome Sanger Institute Scientific Operations: Sequencing Operations are listed here: https://doi.org/10.5281/zenodo.12165051.

Members of the Wellcome Sanger Institute Tree of Life Core Informatics team are listed here: https://doi.org/10.5281/zenodo.12160324.

Members of the Tree of Life Core Informatics collective are listed here: https://doi.org/10.5281/zenodo.12205391.

Members of the Darwin Tree of Life Consortium are listed here: https://doi.org/10.5281/zenodo.4783558.
