# Supplementary material for: The genome sequence of the virgin bagworm, Luffia ferchaultella (Stephens, 1850)
Source: Wellcome Open Res. 2025 Feb 26;10:108. [Version 1] doi: 10.12688/wellcomeopenres.23768.1 (PMC12391867; doi:10.12688/wellcomeopenres.23768.1)
Supplement: Peer review history

# Reviewer report: Reviewer response for version 1

**DOI:** 10.21956/wellcomeopenres.26216.r127278

**Published:** 2025-09-05

**Authors:** Annabel Whibley

**Affiliations:** 1 Grapevine Improvement, Bragato Research Institute, Lincoln, New Zealand

In this Data Note, Whiteford and colleagues report the reference genome assembly and annotation of *Luffia ferchaultella*, commonly known as the virgin bagworm. The manuscript highlights the particular interest in this sample as potentially a derived parthogenetic form of *L. lapidella*, and the comparative genomics of this system will be an interesting follow-up.

 The genome assembly is of high quality with methods and reporting following standard Darwin Tree of Life templates. The links to publicly available  datasets are functional, and report of methods and metadata is comprehensive and well-structured. This is the first Data Note I have reviewed that uses the ultra-low input methods and PCR to obtain the backbone HiFi sequence reads and it is encouraging to see the assembly QC stats remain excellent despite the additional interventions required for low-input datasets.

Are sufficient details of methods and materials provided to allow replication by others?

Yes

Is the rationale for creating the dataset(s) clearly described?

Yes

Are the datasets clearly presented in a useable and accessible format?

Yes

Are the protocols appropriate and is the work technically sound?

Yes

Reviewer Expertise:

Genomics, Bioinformatics, Evolution

I confirm that I have read this submission and believe that I have an appropriate level of expertise to confirm that it is of an acceptable scientific standard.

# Reviewer report: Reviewer response for version 1

**DOI:** 10.21956/wellcomeopenres.26216.r127279

**Published:** 2025-08-27

**Authors:** Panagiotis Ioannidis

**Affiliations:** 1 Foundation for Research & Technology - Hellas, Crete, Greece

This manuscript describes the genome sequencing, assembly and annotation of the Luffia ferchaultella genome. The assembly quality is good with the vast majority of the assembly being found in 31 chromosome-scale scaffolds. Moreover, BUSCO scores, blob plots etc agree a high quality assembly.

 I only have two comments:

 1) Figure 1 must be better than the one currently provided by the authors. I understand that this larval protective bag is very characteristic for this species, but it cannot be the only photograph provided because it's not easy to understand what exactly is shown. I suggest that the authors (a) provide a more detailed description of the figure showing the larval protective bag, and (b) add photos of other developmental stages as well.

 2) It is very nice to see that this genome was also annotated with the Ensembl Rapid Annotation pipeline. However, the quality of the gene set wasn't evaluated whatsoever, which is very problematic. As I have mentioned in my other reviews in this journal, it should be "obligatory" to report the BUSCO scores for the gene set as well (i.e. in addition to the assembly). This is very important so that the potential users know how much they can trust the gene set. If, for example, the gene set has a Complete BUSCO score of 85%, while the corresponding score for the genome assembly is >95%, then something went terribly wrong during genome annotation.

Are sufficient details of methods and materials provided to allow replication by others?

Yes

Is the rationale for creating the dataset(s) clearly described?

Yes

Are the datasets clearly presented in a useable and accessible format?

Yes

Are the protocols appropriate and is the work technically sound?

Yes

Reviewer Expertise:

insect genomics, bioinformatics

I confirm that I have read this submission and believe that I have an appropriate level of expertise to confirm that it is of an acceptable scientific standard.

# Reviewer report: Reviewer response for version 1

**DOI:** 10.21956/wellcomeopenres.26216.r127281

**Published:** 2025-08-22

**Authors:** Yu-Feng Huang

**Affiliations:** 1 National Chung Hsing University, Taichung, Taiwan

This genome report presents a chromosome-level assembly of Luffia ferchaultella, leveraging PacBio HiFi long-read sequencing for primary assembly and Illumina Hi-C data for scaffolding. The study is well-aligned with the standards of the Darwin Tree of Life project and includes comprehensive assembly metrics (e.g., scaffold N50, BUSCO scores, QV). The assembly provides a valuable genomic resource for studying parthenogenesis, sex chromosome variation, and host-endosymbiont interactions in Lepidoptera. However, several aspects would benefit from further clarification or discussion to enhance the scientific value and reproducibility of this work.

 Major:

1. Hybrid Assembly Consideration
  The authors relied primarily on PacBio HiFi reads for the initial genome assembly and used Hi-C data for scaffolding. While HiFi reads offer high accuracy and long-read continuity, short-read Illumina data, which are often used for polishing or hybrid assembly strategies, were not employed in the contig-level construction. Given the known strengths of combining long- and short-read sequencing for error correction and resolving low-complexity or repetitive regions, the manuscript would benefit from a brief discussion on whether hybrid assembly or short-read-based polishing (e.g., with Illumina) was considered or intentionally omitted—and why.
2. Clarification on the Role of RNA-Seq Data
  The authors mention the use of RNA-Seq data for genome annotation and state that 12,416 protein-coding genes were identified through Ensembl’s Rapid Release pipeline. However, the manuscript lacks details on how RNA-Seq data specifically contributed to the annotation. It would be helpful to clarify the following:
  - Was RNA-Seq used directly for transcript alignment and gene model prediction?
  - What proportion of annotated transcripts were supported by RNA evidence?
  - Were RNA-Seq reads assembled into transcripts prior to alignment, or were raw reads aligned to the genome directly?
    Providing this information would strengthen the transparency and utility of the annotation pipeline described.

Are sufficient details of methods and materials provided to allow replication by others?

Yes

Is the rationale for creating the dataset(s) clearly described?

Partly

Are the datasets clearly presented in a useable and accessible format?

Yes

Are the protocols appropriate and is the work technically sound?

Yes

Reviewer Expertise:

Bioinformatics, genome assembly and gene annotation, cancer biology

I confirm that I have read this submission and believe that I have an appropriate level of expertise to confirm that it is of an acceptable scientific standard, however I have significant reservations, as outlined above.

# Reviewer report: Reviewer response for version 1

**DOI:** 10.21956/wellcomeopenres.26216.r127282

**Published:** 2025-08-19

**Authors:** Andrew Mongue

**Affiliations:** 1 Department of Entomology and Nematology, University of Florida, Gainesville, Florida, USA

Darwin Tree of Life has produced another insect genome assembly. As usual, the sequencing approach and final assembly are high quality. These genome notes are designed to be brief, but even so I appreciated the highlighting of unique parthenogenetic biology and presence of Wolbachia here. The data and context provide a good foundation for future research.

Are sufficient details of methods and materials provided to allow replication by others?

Yes

Is the rationale for creating the dataset(s) clearly described?

Yes

Are the datasets clearly presented in a useable and accessible format?

Yes

Are the protocols appropriate and is the work technically sound?

Yes

Reviewer Expertise:

Entomology, evolutionary genomics, sex chromosomes

I confirm that I have read this submission and believe that I have an appropriate level of expertise to confirm that it is of an acceptable scientific standard.
